# Experiences of mental health support services in a community sample of parents of children with special educational needs and disabilities

**DOI:** 10.1371/journal.pmen.0000695

**Published:** 2026-08-21

**Authors:** Astrid Erica June Bowen, Laura Fox

**Affiliations:** The University of York (The Department of Education), York, England, United Kingdom; University of Strathclyde, UNITED KINGDOM OF GREAT BRITAIN AND NORTHERN IRELAND

## Abstract

Parents of children with Special Educational Needs and Disabilities face challenges on top of everyday parenting, placing them at higher risk of mental health difficulties. However, this could be alleviated by appropriate and timely targeted mental health services. This study examined the experiences of mental health and mental health services in a community sample of parents living in and around York, England. Qualitative data from 66 survey respondents (89.4% female) and 12 interviewees (67% female) were thematically analysed. Results indicated parents experienced numerous challenges related to navigating complexity in their day-to-day lives and in accessing support for their children. Systemic limitations in service accessibility were identified, with participants relying on informal networks and charitable organisations for support due to insufficient or inappropriate formal provision. For some, services were experienced as ineffective or harmful. The absence of appropriate external support for children placed a substantial burden on parents, contributing to psychological distress, isolation, and parents neglecting their own needs to provide for their children. Participants called for more integrated, strengths-based, and family-centred approaches, particularly flexible parallel provision for parents and children. These findings highlight the need for systemic reform to ensure that support services are accessible, joined-up, and responsive to both child and parental needs.

## Introduction

Parenthood is a natural source of many joys and stressors, but the additional challenges of raising children with special educational needs and disabilities (SEND) can negatively impact parental mental health [1]. Insufficient support provision for children with SEND, however, is common across the United Kingdom (UK). Parents often have to fight for adequate support, which is not only time-consuming but can have a detrimental impact on their mental health [2, 3]. This is concerning, given the impact parental mental health can have on children and the wider family unit [4]. However, despite the additional stressors involved in navigating the complexities of SEND family life, all being obstacles to accessing mental health support, services targeted at or tailored for parents of children with SEND are sparse, and our understanding of parents’ experiences of navigating the services that are available is lacking. This study aims to explore these experiences in a community sample of parents living in York. England.

For the purpose of this article, the authors adopt the definition of mental health provided by the World Health Organisation (2025): “Mental health is a state of mental well-being that enables people to cope with the stresses of life, realize their abilities, learn and work well, and contribute to their community. It has intrinsic and instrumental value and is a basic human right”. The authors see mental health as a continuum, and that individuals may be at risk of developing mental health disorders, such as depression or anxiety, which may require treatment and may impact on an individual’s wider well-being, which encompasses the way they feel about their wider quality of life and their ability to contribute to society.

SEND is a broad umbrella term which covers all children who require additional support based on physical, social-emotional, cognitive, or communication needs. Currently, in England, over 1.8 million children (20%) have SEND [5], with prevalence rates being similar globally (15% [6] In the UK, the SEND code of practice emphasises the importance of practitioners working in partnership with parents to identify appropriate means of support for them and for their children [7]. However, lack of availability of services to meet these needs has a knock-on effect on the mental health of parents, with accounts illustrating the stresses that not having access to an appropriate educational setting or support for their child introduces into their daily lives [8].

Studies have consistently identified that parents of children with SEND are at a heightened risk of developing mental health difficulties. A UK-wide survey by Contact a Family [9] revealed 72% of parents of children with SEND had experienced symptoms of mental ill health such as anxiety or depression. This figure is 4.6 times higher than the NHS-reported rate of one in six adults (15.7%) in the general population who were experiencing common symptoms of mental illness in 2014 [10]. Reasons for these higher rates appear to be related to the stresses and concerns of caring for a child with additional needs, including the associated financial burdens, and loss or reductions in opportunities and social activities for the parent [11, 12]. Importantly, the quality of the parents’ social support network, including family supportiveness and the relationship with a partner or spouse, seems to play a role in mitigating or exacerbating the experience of stress and mental illness symptoms [13, 14]. This suggests a need for support services to work at the family level and to take into account the multifaceted challenges parents encounter, rather than working with a single parent in isolation.

In addition, the challenges linked to managing child behavioural difficulties seem to be an important factor for mental health challenges in parents. Lecavalier et al. [15] found that children’s behavioural challenges, especially conduct problems, linked to parental stress, indicating a transactional cycle where each worsens the other over time. Furthermore, Blacher and McIntyre [16] found that controlling for behavioural symptoms, a child’s diagnosis did not add unique variance to parent mental health, implying severity and diversity of behavioural issues are more crucial in predicting parental stress than diagnosis type. This suggests that timely interventions are likely to be vital to interrupt and de-escalate worsening mental health trajectories over time, and such support cannot rely solely on a child’s diagnosis, implying that mental health support for parents will need to be tailored and individual, based on their personal situations.

There are also specific sub-populations of parents of SEND children who may be more vulnerable to mental health difficulties. Elevated depression scores were seen in mothers of autistic children and/or intellectual disability relative to fathers and non-SEND controls, with single mothers particularly vulnerable to greater depressive symptoms [17]. Furthermore, research shows that many children with SEND are likely to have family members with similar traits or diagnoses. Studies suggest that ADHD has a heritability of 70–80% [18], and autism a rate of 40–80% [19]. Autistic mothers have reported finding motherhood more isolating and less rewarding than non-autistic mothers, and expressed that they struggle to seek support, and feared disclosing their own diagnosis would alter professionals’ perceptions of them [20]. This further adds to the complexity of family life, as parents may not only be supporting multiple children with SEND, adding additional demands onto their parenting load, but may also be neurodivergent or have additional learning needs themselves, highlighting the need for integrated support services which support SEND families holistically and are neurodiversity-affirming. Despite the wide range of SEND, much of the research on parent experiences of SEND has been done with parents of autistic children, and while important, research exploring the wider experiences of parents whose children have a range of needs is lacking even more so. With much research focusing on one specific need, the voices of parents with children with complex or multiple needs are often missing.

Accessing support for mental health remains one of the most significant barriers identified by this community. Even when parents recognise their own need for support, they are frequently met with systemic hurdles that render help inaccessible, resulting in many only seeking out mental health support when the severity of their needs is high [21]. In the UK, the National Health Service (NHS) provides universal, free healthcare. General practitioners (GPs; family doctors) serve as the primary point of contact for concerns, including mental health difficulties, who then make referrals to specialised services when necessary. Although access to the NHS is free, improving equity in access to services often results in long wait times to access appropriate support [22]. Access challenges for parents of children with SEND extend beyond these long wait lists, with parents reporting that even accessing initial meetings is unattainable due to childcare challenges [21]. Understanding these complexities at a local level can support the development of best practice to improve access to services, which may then translate into wider policy change surrounding access requirements.

For those who do access support, it is rarely reported as accessible or appropriate to parents’ needs. Many express feeling unsupported around the time of their child’s diagnosis [23], with 78.7% of mothers of autistic children reporting clinically significant depressive symptoms during the week following diagnosis of their child, with 37.3% persisting at follow-up an average of 1.4 years later [1]. Similarly, in Crane et al. [24], parents of autistic children reported that professionals, especially GPs, appeared to demonstrate a lack of autism awareness during the autism diagnosis process, increasing anxiety at an already stressful time. The evidence here suggests that support for parents’ mental health must be available from the earliest point of their children’s diagnosis process if it is to be successful, and provided by trained individuals, yet it is clear that this is not consistently available at present. The quality of formalised support services has also been identified as an important protective factor against stress in parents of children with SEND [25]. This is theorised to operate through mechanisms including improved coping skills, which have been identified as a moderating factor in stress responses to child behaviour [26]. Although this and other past literature suggest that targeted support can reduce mental health difficulties in parents of children with SEND, there appears to be limited targeted provision for parents [24,27]. Gaining a locally sensitive understanding of parents’ experiences of the current limited provision, and the types of provision they would like to see, will help inform better and more acceptable mental health services for this population.

Together, the above suggest a clear need for tailored and flexible support. However, in a scoping search of services in the York area for the current study, the only tailored mental health support for parents of children with SEND identified was a variety of support groups and social media forums for parents to connect with and support each other, and as parents frequently report not having time to pursue their own activities, it may be more difficult for them to access these types of services (e.g., [3]. It is also important to note that while support groups are a very common means of promoting good mental health for parents of children with SEND, parents have not rated this kind of service as highly as tailored professional support services in terms of perceived helpfulness [23]. Furthermore, the extent to which local health services, such as counselling, currently meet the unique needs of parents and caregivers of children with SEND was not clear based on this search. Therefore, it appears there may be room to develop and improve the range and specificity of mental health services currently available to parents of children with SEND living in and around York, and by including the voices of local parents, we hope to advocate for a shift in policy and practice that recognises this need at a local level, which may then inform more national-level services.

### The current study

Previous research clearly shows that parents of children with SEND are not only at risk of poorer mental health, but that they have restricted access to suitable support services; however, current research focuses predominantly on small groups of parents supporting specific SEND (e.g., autism). The current study aims to expand this knowledge by exploring the experiences of parents with children who have a wide range of needs in more detail in the specific context of York. York serves as an excellent micro-system for studying parents’ mental health experiences when caring for children with special educational needs and disabilities (SEND), providing insights that are unique to York and that can be directly applied across the UK. Systemically, parents in York navigate the standard institutional frameworks and structural pressures characteristic of English local authorities and NHS integrated care systems, including Education, Health and Care Plan (EHCP) pathways, multi-agency coordination, and delays in specialised services. Furthermore, whilst York’s socio-economic landscape mirrors broader national health divides, featuring pronounced variations between affluent suburban wards and localised pockets of socioeconomic deprivation, around 5% of York’s population live in areas ranked amongst the bottom fifth in England [28], placing areas of the city in the NHS Core20Plus5 [29]. This has led to wide inequalities in healthcare access, and allowing the exploration of how local support networks and community support in different wealth areas may influence parental mental health and access to support.

While localised commissioning structures may vary slightly by region, the fundamental stressors and systemic barriers experienced by families raising children with special needs remain structurally consistent across England. Consequently, findings from York can yield scalable, nationally relevant insights capable of informing family-centred mental health policies, early intervention strategies, and targeted support frameworks for SEND caregivers across the UK. Therefore, using a combination of survey and interview methods, the current study aimed to develop an understanding of parents’ and caregivers’ mental health and their experiences of navigating services in the City of York and the surrounding areas, including whether current services are perceived to be adequately meeting their needs, and how parents believe that service provision could be improved. The research questions were:

- How do parents/caregivers of children with SEND living in and around York perceive their own mental health support needs and their experiences of accessing this support?
- What, if any, are the needs/barriers to support faced by local parents of children with SEND in relation to mental health and well-being?

## Method

### Design

This was a mixed-methods exploratory study with two components: 1) an online survey containing a combination of quantitative and qualitative responses; 2) follow-up semi-structured interviews.

### Participants

Participants were recruited via opportunity and snowball sampling through contacts with local schools and charities, social media advertisements, parenting groups and by distributing physical and digital flyers. Links to the survey were distributed widely to reach as many parents of children with SEND in York and the surrounding areas as possible. Some interview participants were recruited via a contact form at the end of the survey, others were recruited through alternative methods, including local schools, charities, and parenting social groups. The two main inclusion criteria for the study were a) participants must be over 18 and a parent of a child with SEND, and b) participants must currently live in or around the city of York, England.

*Survey*: 66 parents responded to the online survey. Demographics of the final sample are presented in Table 1.

**Table 1:** Demographics of Survey Respondents.

Demographic Variable	*n*	% / M (SD)
*Gender*		
Man	7	10.6%
Woman	59	89.4%
**Total (N)**	66	100%
*Age*		
Overall	—	42.5 (9.7)
Male	—	42.23 (9.7)
Female	—	41.9 (9.7)
*Marital Status*		
Cohabiting	8	12.1%
Married/Civil Partnership	47	71.2%
Separated	4	6.1%
Single	6	9.1%
Widowed	1	1.5%
*Parent SEND Status*		
No SEND	46	69.7%
Suspected SEND	10	15.2%
Unsure	2	3.0%
Yes (Diagnosed)	8	12.1%

*Note.* M = Mean; SD = Standard deviation; SEND = Special Educational Needs and Disabilities. Percentages may not sum to 100% due to rounding.

**Fig 1:**
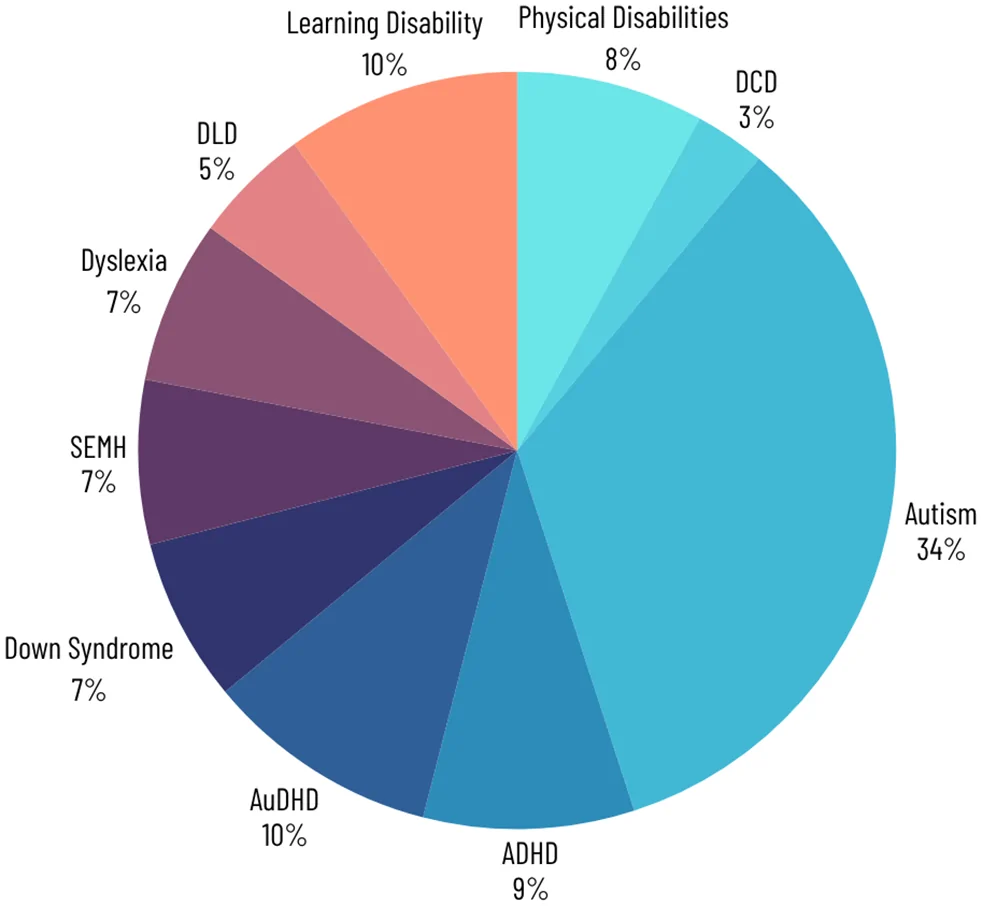
Breakdown of Parent-Reported Child Diagnoses. Note. ADHD = Attention Deficit Hyperactivity Disorder; AuDHD = Autism and Attention Deficit Hyperactivity Disorder; DCD = Developmental Coordination Disorder (Dyspraxia); DLD = Developmental Language Disorder; SEMH = Social Emotional Mental Health.

Children of survey participants were aged between 0–24 years (*M* = 11.3 years, *SD* = 6). The average age at which children received a diagnosis was 7.2 years. Fig 1 displays the breakdown of child diagnoses, derived from parents’ reports of their children’s diagnostic status. Including the AuDHD category (a combination of Autism and ADHD diagnoses), 44% of children had co-occurring diagnoses.

*Interviews*: 12 participants who had either previously completed the survey or been recruited through other means were interviewed, of whom 8 (67%) identified as women. For those not recruited through the survey, the same selection criteria were used. Targeted recruitment was conducted through a local support group for men to ensure a more balanced gender split in the interview study. The age range of participants was 36–57 years (*M* = 49, *SD* = 7.3). The mean interview length was 50 minutes (*SD* = 18). Demographic information and pseudonyms for interview participants can be found in S1 Table.

## Materials

The survey used a novel questionnaire developed by the team to assess demographic characteristics of parents/caregivers and their children, their awareness and usage of mental health services in the York area, and their experiences of mental health challenges in relation to their status as parents/caregivers of children with SEND.

A schedule for semi-structured interviews was devised to go into greater depth on parents’ experiences of mental health difficulties and support services in the York area. This was developed based on common responses to the survey. Participants were invited to discuss barriers to mental health support and to share ideas with a view to providing recommendations for improving existing services or introducing new services.

All materials for the study can be found on the project’s OSF page.

### Procedure

Participants accessed the anonymous online survey between October and November 2024, remotely via Qualtrics. They were provided with an information sheet explaining the aims of the study and how their data would be used. Those interested in participating in follow-up interviews followed a second link to register their interest in order to avoid de-anonymising the surveys. Given the sensitivity of the topic, support links were provided to parents in case anything discussed in the survey became upsetting.

Interviews were conducted online through video conferencing software and carried out by the first author. One interview was conducted in person at the university due to personal preference of the interviewee. Participants signed a consent form prior to the interviews, which included signposting to relevant support groups. Participants were also given the opportunity to ask any questions before the recording began and had the option to comment on their transcript for up to 4 weeks after their interview, after which time the data would be anonymised. Audio recordings were transcribed verbatim using a university-approved transcription service.

### Analysis

Descriptive statistics were produced on demographic and Likert question responses for the survey. Open-ended survey responses and interview transcripts were analysed using Braun and Clarke’s (2021) method of reflexive thematic analysis to identify shared themes relating to parents’ experiences of mental health and mental health support services. Coding for survey (second author) and interview data (first author) was conducted separately; codes were mapped onto each other and iteratively refined to identify overall themes that were shared across the dataset. Analysis for the study was pre-registered on the OSF.

### Ethical considerations

Ethical approval was received from the University of York’s Education Ethics Committee before the study commenced (FC24/4). Participants provided written informed consent. All participants were provided with links to support groups in their information sheet in case discussing their experiences was distressing.

### Reflexivity statement

Both authors have professional and personal experiences with parents of children with SEND, and of children and young people with additional needs themselves, which may have influenced the analysis. Both authors have experience working with children with additional needs in school settings, and families of children with SEND in community settings. The second author also has close personal ties with several parents of children with SEND. These experiences may make both authors particularly aware of the challenges faced at home and school level, albeit from an outsider perspective. However, great care was taken to stay as close to the participants’ stories as possible, with both authors meeting regularly to discuss the analysis.

## Results

**Fig 2:**
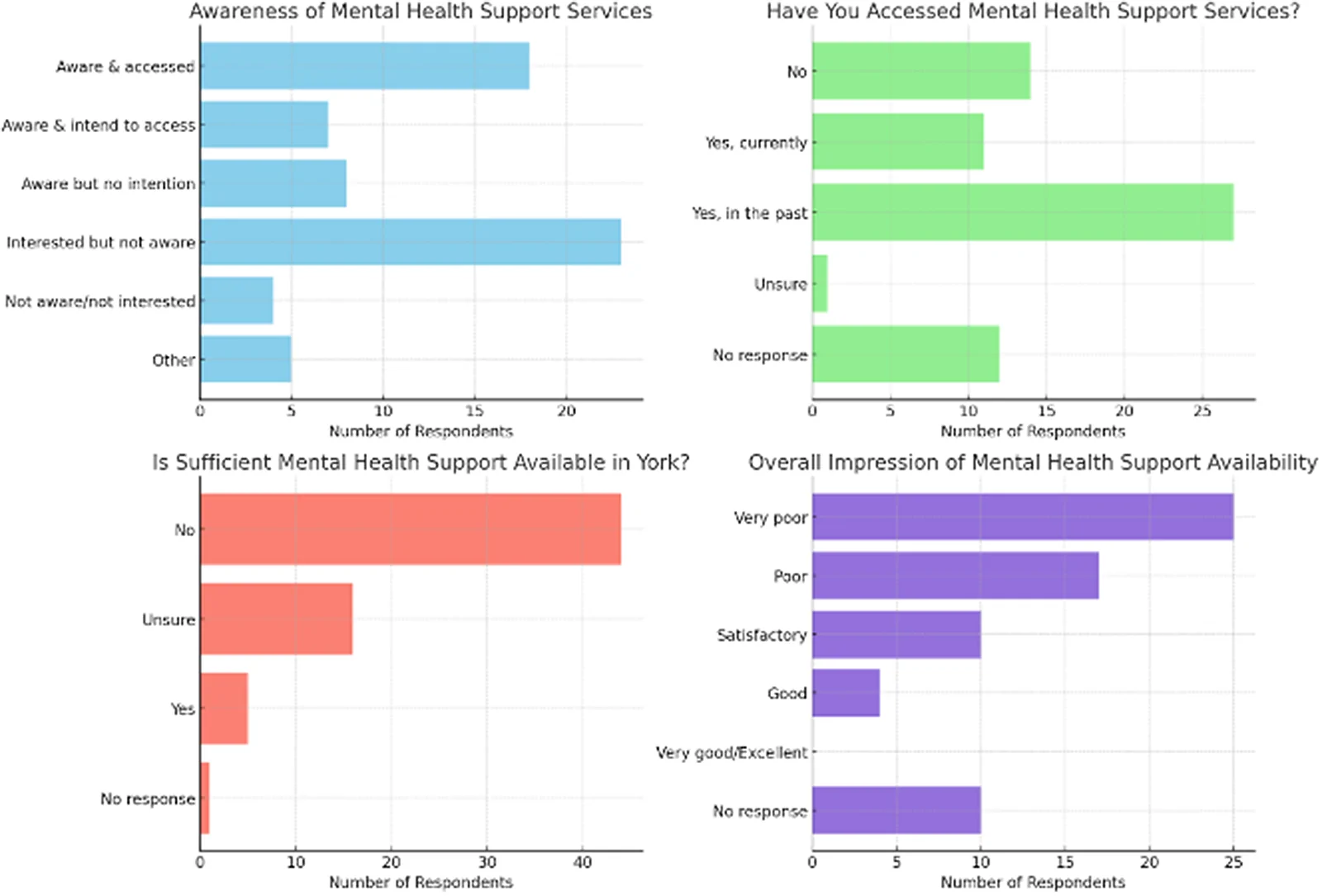
Participant responses regarding awareness, access, and impressions of mental health service availability.

Fig 2 presents descriptive statistics for survey respondents regarding their levels of awareness of mental health support services, whether they have accessed or intend to access such services, whether they believe sufficient mental health support is available in York, and their overall impression of the availability of mental health support services. Sixty-eight per cent of respondents did not feel that there was sufficient mental health support available for parents of children with SEND in the York area, and 75% believed that service availability was either Poor or Very Poor. When asked directly, no interview participant reported that mental health services in and around York currently met their needs.

Three overarching themes were identified across survey and interview data. Participants from the interview section of this study are referred to using pseudonyms; questionnaire participants are identified by a participant ID number due to the larger number of responses.

## Theme 1: Navigating the complexity of SEND family life

Parents spoke about the multiple layers of complexity that surrounded supporting a child with SEND, including complexities of family situations, the diversity of their child’s needs, and the impact this had on their mental health. For many, the family unit involved multiple individuals with additional needs, and the additional stressor of navigating obtaining a diagnosis and support services for children played a key factor in mental health challenges.

### The complexity of family life

Parents reported ‘burnout due to constant competing demands’ and ‘relentless challenges’. They described difficulties and frustrations with the demands placed upon them, including balancing the needs of children with other areas of life, such as work, friendships and self-care: ‘So often things need immediate response, but I have this from both kids so I am pulled both ways, and then being told I need to take time for myself!’ (QP1037). Grace, a single parent, discussed the difficulty of applying commonly-advised strategies for individual self-care, as putting the child’s needs first is the priority for parents: ‘You can’t just go like “Well, I’ll just go out for a walk.” [...] I’ll set some time aside for self-care just as soon as the person that’s completely dependent on me magically stops being dependent on me.’

Many parents were supporting multiple children with SEND, which was reported to put further stress on family life, often at the cost of parents’ mental health:

‘Not being able to leave my house on my own with them [be]cause double disability buggy[ies] is like trying to track down a unicorn and they can’t walk safely so have to have 1:1 adult support outside of the house. There’s so much I could write that can affect my mental health through being a SEND parent, it’s a very big mental load to take on, and if you don’t have strong mental health beforehand, it definitely takes a battering’ (QP1065)

Here, the logistical challenges of having children with additional needs often limited parents’ ability to go about daily tasks. As a result, parenting a child with SEND was often characterised as an isolating experience, as parents were not able to participate in activities that other families do:

‘It sounds really dramatic, but I suppose I’ve lost part of my personality. We can’t go to the restaurants I want, because [son] won’t eat that food. We can’t go on the holidays that I would prefer or my husband would prefer, because [son] won’t tolerate those type of holidays. We can’t do the day trips that we really fancy because he won’t tolerate the car journey or the type of activity when you get there, whereas friends of ours go, “Well, they’ll just have to do it, won’t they?” And it’s, like, “No, because he has a meltdown and it’s horrible and then we have to come home, so no, actually, that’s not how it works.”’ (Rachel)

Alongside feelings of isolation, it was clear that the role of carer was often placed on one parent (most frequently mothers), and this had led to some reporting a loss of self-identity: ‘I often find it hard to enjoy doing anything and crave a time when I can be myself again’ (QP1002). This may suggest that even for those parents who are not explicitly speaking of isolation and loneliness, the demands of parenting could have a lasting impact on self-identity and, in turn, mental health.

The demands of the parenting role were inextricably linked with parental mental health: ‘My mental health is linked to all of the demands placed on me, of which [participant’s son] is the biggest demand, and a relentless demand at that’ (Rachel). In addition to caring demands, participants reported receiving physical and/or emotional abuse from their children: ‘It was like an abusive relationship that I couldn’t leave’ (Julia); ‘I have been subjected to traumatising episodes of violence from my child’ (QP1005), which had had lasting impacts on their daily lives and mental health. For some, this was an ongoing challenge that negatively impacted multiple areas of parents’ well-being: ‘My daughter has had prolonged periods (i.e., years) of being physically and verbally aggressive towards me and this is exhausting, distressing and completely destructive to your self-esteem’ (QP1033).

For those who had reached out for support, the complexities above often meant they were unable to attend doctors’ appointments. When support was offered over the phone, it was often at inappropriate times:

‘Services that understand needs of carers especially around challenges with when they can get to appointments, that caring responsibility might mean late cancellation- not just 3 strikes and you’re out. As previously stated often there is no negotiation on convenient times. I once had a GP ring (at a time I said I was not available) to talk about suicidal thoughts, just as we were setting off [on the] school run. I ended up trying to speak locked in the bathroom with daughter screaming at the door as [we] were going to be late. Ring when you say you are going to! Always check it’s a good time - my SEN kids don’t care about my crisis.’ (QP1038)

Finally, many discussed how navigating their child’s diagnosis had led them to question their own neurodiversity. This added to the family complexity, with some speaking of how past traumas surfaced as a result of the process, suggesting that many families might face several layers of challenges when reaching out for mental health support:

‘I have also been for an initial ADHD and autism assessment and am on the waiting list for full diagnosis. This is through right to choose. Going through this for my son has raised lots of red flags and trauma from my childhood which again is difficult to process’ (QP1018)

### Fighting for support and the impact on parental mental health

It became clear that parental mental health and child services are deeply interconnected; getting SEND services right for children appeared to be related to improved mental health for parents: ‘If my daughter’s happier and goes to school, I will be happy [...] I just want support for [daughter], focus on that, because then that will offer me support by her getting that’ (Julia). Long waiting times for SEND services exacerbated problems until parents required crisis support, which in turn was characterised as ineffective. For example, Emma, who rang a crisis support line, was advised to call the police for her son’s behaviour: ‘He’s eight years old, I don’t really want to be ringing the police on my eight-year-old son; that would freak him out. So yeah, I don’t really get the feeling that there is much mental health support for parents.’

The process of accessing support for children’s SEND needs was described as extremely long, complicated and challenging: ‘It’s been fight, fight, fight for three years’ (Mary). The amount of form-filling and the complexity of forms required to access support were frequently referenced: ‘We’ve filled out *War and Peace* worth of documentation. [...] I feel like I’ve filled the same form in about 20,000 times already’ (Emma). There appeared to be agreement across interview and survey participants that parents are expected to compensate for missing or ineffective services: ‘I’d say the past three years has basically been spent, for myself, filling out lots of paperwork and battling systems and trying to be a specialist in speech and language therapy, occupational therapy, autism’ (Mary). James described the additional workload of managing the services on top of regular care:

‘It does all seem to end up landing back on us [...] You can’t look after him and also try and handhold all the services that are really meant to help you. You know, it’s just not doable. [...] I’ve got a full-time job and lots of stuff I have to be dealing with outside of that and it is like having another job.’

Although some parents were navigating these demands alongside a full-time job, a large proportion of participants reported having to change jobs or reduce working hours to manage the care of their children and liaise with different agencies, which led to mental health challenges:

‘I’ve had to give up my job due to insufficient wrap-around care and my son’s high needs. So I’m now only working 4 hours a week. It can put you in an impossible financial situation. We’ve had no help with DLA or money paperwork. I’ve had to do it on my own. It’s been really hard. Constantly feeling in fight or flight mode; I’ve lost my job, had multiple burnouts and breakdowns and become very shut off from the world’ (QP1018)

The lives of parents of SEND children are multifaceted and complex. Not only do parents have additional caring needs at home, but it is evident that these stressors extend to navigating child support, managing relationships, and juggling home/work commitments. Those supporting parents will need to be attuned to the multiple layers of complexities both for children and within families, and how this might impact parental mental health, providing sensitive and flexible support avenues.

## Theme 2: Fragmented parental support can cause more harm than good

Parents spoke of the support they had experienced in relation to their mental health. The majority of those who received mental health support reported that they were using or had used private counselling services due to long waiting times and limited support through the National Health Service (NHS). Interview respondents reported that they were not aware of the existence of targeted mental health support services for parents of children with SEND outside of some specific training courses for certain disorders (especially autism) and charity-run initiatives.

Several respondents discussed how stigma surrounding their role as a parent of SEND children created a sense of judgment and feared that seeking out help could result in their children being taken away, hindering their ability to either reach out for support or take advantage of what services were offered:

‘Ultimately the only thing that is offered to you is parent courses. Now when you are in crisis, when somebody offers you a parents’ course, it’s like a kick in the teeth. It’s like a kick in the guts because it’s the implication that you’re a shit parent. If you would only go and do a parents’ course and learn how to do it properly, you would do a better job.’ (Julia)

Others attempted to access support via peer group meetings. While support groups were characterised as valuable, especially due to the social isolation that parents experienced, support groups that were open to everyone with no professional support available sometimes left parents feeling responsible for helping others and neglecting their own needs. For example, James reported his wife’s experience of attending an informal support group:

‘She ended up talking to another parent and actually the situation the parent was in at that particular moment in time was a lot worse than ours. You know, they’re talking self-harm, suicide attempts, all kinds of things. [...] My wife actually ended up offering support to them rather than getting any help herself. And so, she never went to another one because she was saying, “Well, it’s ridiculous. I’ve been there. I went for an hour. I got no help for myself.” [...] She actually came back quite upset from it and in a worse state than she would have been had she not gone.’

Here, attending the group created the opposite effect than intended, negatively impacting members’ mental health and likely increasing their emotional burden.

For those groups that were more formalised, they were characterised as too broad or too narrow in scope, with some parents calling for support for those outside of the most commonly supported neurodevelopmental conditions:

‘[Support] needs to be more than just for parents of children who are ND - [it] feels like the provision that I’m aware of is very skewed towards parents whose kids have autism, ADHD etc; awareness of specific challenges faced by parents of children with SEND but not just those who kids are ND’ (QP1007).

This was echoed across participants, who described the focus of the support offered to them as being too general, and/or the people delivering the support did not have sufficient understanding of what it was like to parent a child with SEND to make the support useful, and a general lack of awareness of what support could be put in place for them. For example, Mary described her experiences of GP support:

‘One doctor even said to me, “You’re a parent, what do you expect?” and I walked out. [...] Another doctor said to me, “What do you want me to do?” and I was, like, “I don’t know, can you tell me what is available? Is there a therapy service or something, is there a drop-in clinic? Like, there must be something.” And they were just, like, “No, I don’t think so.”’

The above indicates that a better understanding of needs is required to match parents and their children with services that best suit them, along with more tailored services for specific profiles of difficulties or challenges that families face. It is clear that both the additional stress navigating services for children and the lack of appropriate, responsive services can harm parents’ mental health, and poor services based on lack of awareness and training can be experienced as harmful. Support that takes this and the complexities of family life into consideration is needed.

## Theme 3: How we need you to help families like ours

Throughout the study, parents discussed how support for their own mental health could be improved. Many parents called for flexible support that was understanding of their needs. Appointments that offered flexible hours and the ability to reschedule without being penalised were key, as were flexible modes of attendance, with many suggesting they should be able to choose between in-person, online, or phone appointments based on their home situations.

Parents expressed a need for specific and consistent contacts so that they could build relationships over time: ‘I don’t want a different person every week, I don’t want an agency, but someone who can become, like, I’d say a professional friend’ (Rachel). For many, this person needed to have an in-depth knowledge of how parenting children with SEND could impact their mental health:

‘I needed someone who really understood what life was like when I’m [in] crisis as a SEND family. It becomes hard to communicate effectively when you are burnt out and there is always this fine balance between being “that parent” and standing up for what your child needs. You don’t want to upset the professionals who are trying to help or working with your child but equally you have to be firm in making sure the provision they need is delivered’ (QP1051)

There was a need for these practitioners to be sensitive to their difficult situations, and ideally had some form of lived experience of what life is like for SEND families:

‘I think you need to have a lived experience and understanding because then I think you can have empathy, as opposed to judgment. And if you go at it with a “I know how hard it is” and I understand that, that’s a completely different footing from somebody who comes in without that, and it does feel judgmental’ (Julia)

Most participants reported that services ended too soon for them, and having a continued point of contact would have helped to alleviate problems after a direct intervention ended. The inclusion of practitioner-led drop-in services was viewed as a much-needed addition to the suggestions above. Due to the lengthy and confusing pathways to diagnosis for children, parents expressed a desire for opportunities to speak with knowledgeable individuals who can answer questions, direct them to appropriate services, and advise them on how to access further support. Although this support was not directly linked to an intervention for parental mental health, it is clear that the burden of accessing children’s services is a large contributory factor to parents’ mental health concerns, and support in navigating this process would be beneficial for them.

Parents across the study were also left frustrated by long GP wait times, or counselling services that had no capacity. This resulted in parents calling for a prioritised pathway for mental health support, recognising that many parents are reaching crisis point while waiting for services for themselves or their children.

Throughout the study, many participants spoke of how getting support right for their children would alleviate the pressures that led to their mental health difficulties, and in turn, reduce the need for them to access mental health services. Accessible respite and practical support, such as access to household management or reliable, trained childcare services, would alleviate some of the considerable challenges parents face. Participants also called for better wraparound care, services that communicated with each other rather than contradicting, and services that considered the holistic needs of the family, not just the child:

‘We were encouraged by our psychotherapist, who’s an adoption expert, to ask for respite, and they said no because there’s no safeguarding risk. Basically [son] was not at risk enough, they’re not bothered about *our* risk, they’re bothered by the child’s risk’ (Rachel)

Furthermore, alongside wraparound care, joined-up support for children, extending into the transition to adulthood, was called for throughout the study. Parents reported that supporting their children did not stop when they turned 18, and highlighted the challenges that accompanied the switch to adult services, which often required multiple repetitions of admin that added pressure on parents and young people:

‘Every separate service you engage with, you have to get your young person’s permission once they’re over 18, or over 16 possibly, to talk to the service on their behalf. So I have that that for PIP, […] But if I want to talk to the GP on her behalf, she’s got to come in with me, which she hates doing, and then talking to people she hates doing. […] And then the same with the ADHD service at the [place], and they’re like, “Well, your young person will need to come on the phone and tell us that we can talk to you” and I do get that it’s really important that they do that. I do get that. But it would be really nice if I could just do that once and then it carried over to all of the other services, so I could sort it at the GP and then it would apply it to the hospital and it would apply to the [place], and you know, it’s all a faff’ (Grace)

Services that could reduce the admin burden during key transition periods, and that take into account the communication needs and reasonable adjustments of young people, would, in turn, support parental mental health.

Despite some challenges with informal support groups (see Theme 2), parents wanted opportunities to meet other parents with lived experiences and to socialise in spaces that felt safe and accepting of their challenges and their children. A setting where parallel provision was in place so children could play in a safe environment while parents had time to socialise. These spaces need to be inclusive of all needs, including children (and parents) with physical disabilities, as it was noted that current social and support groups were catered towards physically able children with intellectual or behavioural disabilities. Julia described a similar model run by a charity for bereaved parents:

‘They have family support days where you can go and take the kids, and the kids do certain activities, and the parents go off and talk to other parents. It’s that kind of community, it’s like bringing everyone together in a positive way, because so much of this can be stressful and negative’

Here, parents called for mental health provision for themselves that was accessible to parents of SEND, supported by trained, consistent professionals who ideally had lived experiences of navigating the SEND landscape. This would be further supported by appropriate and timely support for their children, which would reduce the emotional, physical, and administrative burden that many parents frequently report.

## Discussion

Using data from 66 community survey responses and 12 interviews, this study identified that parents of children with special educational needs and disabilities (SEND) have complex needs across multiple levels that make them more prone to mental health difficulties and create barriers for service access, and that changes to children and adult services are needed to support good mental health across this population.

Firstly, it is clear that parental mental health is intrinsically linked to the complexity that arises from the diverse needs of family circumstances (including those that have SEND themselves or multiple children with SEND), and the emotional and logistical demands of caregiving [23], and that this is a trend that goes beyond the local area and is evident across the United Kingdom (UK) [30]. This shows that stressors for parents in this study align with those nationally and that suggestions for support may be transferable nationally. Deficits in support for children forced parents to spend time and energy applying for support. This often came with financial implications, exacerbating stress and worsening mental health. This is echoed across the UK [31, 32], suggesting that these financial losses are a major source of stress for parents and families nationwide, not just in this geographically limited sample. Mental health support should include guidance on applying for benefits or seeking financial support for children’s care to help alleviate this stress when suitable local authority-provided care is unavailable.

Parenting SEND children also presents unique sources of stress, such as managing the severity and diversity of behavioural issues (Blacker & McIntyre, 2005), and services for children need to be there to alleviate emotional and logistical burdens for parents, rather than adding further workload and complications. Disconnected and overly specific diagnostic pathways prevented many parents from accessing timely support, and long waiting times appeared to be a contributing factor to distress. This is in agreement with Starkie [32], who found that parents were persistently having to fight to access assessments of needs and that this had a knock-on effect on the wider well-being of the entire family. Post-diagnosis services left parents feeling unsupported and unable to cope; services were criticised for placing too much emphasis on a diagnostic label (e.g., autism), making access difficult or inappropriate for many families. Services should provide resources and support for the particular challenges that parents of SEND children face, rather than focusing on educating about the broad features of diagnostic labels. This would align with growing interest in moving towards transdiagnostic approaches to assessment and support that focus on defining and meeting the needs of cognitive and behavioural profiles rather than labels suggested by [33], and with the proposed move to using ‘Areas of Development’ in the recent SEND reform consultation (Department for [34]).

Improved support for children transitioning to adult services and for those at other key transition points is clearly warranted and would alleviate stress for parents. The concern about the longer-term need for care and its impact on both parents and children has been raised recently in a country wide study, with thoughts about what will happen to care when parents are no longer around to advocate for their children being a source of extreme worry [31]. Many children with SEND will require lifelong support, so these findings emphasise the importance of personalised planning that adapts as children grow into adults, and support for parents and the wider family at key transition points. Long-term care for children, including decision-making around residential care during adulthood [35], is emotionally complex and not necessarily a pressure that parents of non-SEND children have. Practitioners who are well-trained or have lived experience are vital in supporting parents in navigating these challenges and, in turn, supporting their mental health.

In addition to a family-focused approach to support, parents desired a wraparound style of services that integrate and communicate with one another, and more practical support, such as childcare provision by trained professionals. While there is a clear need for this, as it would reduce the administrative burden on parents, it would require improvements in data capture and data sharing between services. Studies have shown that blockchain can improve healthcare services by enabling the secure sharing of sensitive data [36], but a wider discussion of this is beyond the scope of the current study. Future studies should explore the role that secure data sharing could play in improving SEND support.

Parents also highlight what did and did not work in the current mental health service. Support was largely characterised as fragmented, inconsistent, and difficult to navigate. As with previous studies [27], parents were mostly unaware of services that were tailored to support their specific mental health needs, with families frequently relying on charitable organisations or peer networks in their absence. Access to support groups was deemed too general, and it was clear that those delivering the support lacked sufficient understanding of SEND families. Peer support groups were commonly accessed in this study, but met with mixed success. Throughout, peer and social support were called for, but this must be done in a more structured and supportive way. Studies have shown that peer support can be effective in improving mental health, but it is most acceptable and successful when co-produced with the population it aims to support and includes guidance from well-trained peer support workers and clinical professionals [37].

One suggestion arising from this study is the need for consistent mental health professionals who have lived experience or sufficient training in SEND. Good-quality relationships have been shown to support good mental health [38], and extending this to relationships with practitioners would likely create a safe and predictable space for parents to access support. Having a consistent point of contact would reduce the need for parents to retell challenging and potentially traumatic past experiences. Consistent professionals who are trained in adapting provision for those with neurodivergence, including communication styles, are also crucial. Many parents in the study alluded to being neurodivergent themselves, which impeded access to support. Parents’ wants in this study align with a recent study on developing accessible autistic ‘SPACE’ (Sensory needs, Predictability, Acceptance, Communication and Empathy) [39]. Integrating these elements into mental health provision would likely help reduce stigma, make individuals feel safe to seek and access support, not just in this population, but in the wider neurodivergent population. Provisions should aim to include parallel, accessible provision for children to aid parents in attending.

Accessing traditional forms of mental health support was a challenge for this cohort, partially because of the complexities of arranging childcare in order to go to in-person appointments. One alternative may be to promote digital mental health support tools. It is unlikely that, given that parents often seek support informally due to the challenges highlighted above, they have information about access to digital tools. Digital mental health care has the potential to make prevention, diagnosis, and treatment of mental illness flexible, cost-effective, and accessible, regardless of location [40, 41]. Tools such as rule-based and generative artificial intelligence (AI) chatbots have been shown to be supportive for those with mental health difficulties [42], and their round-the-clock availability and accessibility via mobile phones allow individuals to obtain help at any time or place, overcoming financial and logistical barriers, which would be beneficial for parents of SEND globally. The limitations of these tools must be clearly explained to users; however, there is limited research exploring their acceptability among parents of SEND or within the wider neurodivergent population. Future research would benefit from further exploration of their potential to support this cohort’s mental health needs and the adaptations needed to make the tools usable for diverse needs.

While participants belonged to a specific local community of SEND parents in and around the City of York, findings appear to align with those from a recent series of workshops with parents and children aimed to capture data on experiences of SEND services at the national level for England [30]. This report highlighted the need for joined-up services, and went a step further down the transdiagnostic route to argue that provision of support should not hinge on the presence of a formal education, health and care plan (EHCP), a change which would make services more proactive and responsive to needs before they have the chance to escalate over time. Furthermore, given the known heritability rate of many neurodivergent disorders and the likelihood of neurodivergent parents, the findings here support the recent independent review of mental health conditions, ADHD and autism, which highlighted the lack of accessible mental health services for this population [43]. Taken together, this suggests that many of the recommendations here likely reflect similar cohorts across the UK, but may not be applicable to countries that operate a mixed-model healthcare system, combining public and private service provision, such as Australia or Canada, as private healthcare insurance likely reduces pressure on public services, resulting in shorter waitlists than the UK. This in itself suggests that mental health service design and education support systems in the UK may, in themselves, contribute to mental health difficulties and delayed mental health support experienced by parents of children with SEND.

One of the key takeaways from this study is the need to redefine what mental health support looks like for parents of SEND children. While specific mental health support services, such as counselling by informed practitioners and peer support groups, have their place, it is clear from these findings that mental health support for parents starts with getting SEND support services right for children. There is also work to be done on what a comprehensive network of support services for SEND looks like and what the impact could be not only on the children but also on their families.

## Limitations

A key limitation of the study is its place-based approach to data collection. Although it includes a good sample of parents’ perspectives, it may not be applicable to other areas with different levels of mental health services or to countries that have different mental health and special education systems. Therefore, care must be taken when generalising the findings from this study. Future research should build on this study to collect a broader range of experiences, supporting more comprehensive mental health planning at a national and global level.

Another limitation is that, although the study aimed to reach as many parents as possible through physical flyers and social media, it remains possible that those with the greatest support needs could not participate due to the additional time involved in the survey or interview. Alternatively, there is a risk that parents who are least satisfied with the existing support structures, or those who have the most difficulty accessing support, were more motivated to participate in the current study, which may further skew the sample. Without more detailed background information on participants, we are unable to identify which bias is more likely. Future work should focus on reducing this barrier by conducting work within the community, preferably in a setting where parents can bring their children if needed, and in a way that facilitates discussions for all parents, regardless of their current or previous engagement with services. Researchers should also offer flexible and multimodal ways to participate to make this easier for all community members.

As this paper focused on parents’ experiences, their suggestions on how future support should be shaped may be limited by the services they are aware of or have accessed in the past. Future work should explore the acceptability of new methods for monitoring mental health, such as those using electroencephalogram (EEG) or deep learning, and how advancements in digital tools may provide mental health support solutions that ease the burden on parents.

Finally, no information on ethnicity or deprivation/socioeconomic status was collected during this study, meaning that intersectionality and links between potential inequalities and the accessibility of mental health services cannot be discussed. Future research would benefit from gathering this additional information to explore which families may need further support accessing services, including exploring child diagnosis type, service awareness and satisfaction.

## Conclusion

A combination of data from a community survey and interviews revealed that parents of children with SEND experience sustained emotional, logistical, and economic burdens which are frequently exacerbated by fragmented service provision and poor accessibility. Parental mental health is intrinsically connected to the well-being of their children, and so, in addition to developing targeted mental health support for parents, there is a need for SEND service reform to prioritise integrated, family-centred approaches that can respond in a timely manner and take a lifelong approach to support that encompasses both the child and their wider family context.

## Supporting information

S1 Table Demographic information for interview participants with pseudonyms. (DOCX)
