# Supplementary material for: Experiences of mental health support services in a community sample of parents of children with special educational needs and disabilities
Source: PLOS Ment Health. 2026 Aug 21;3(8):e0000695. doi: 10.1371/journal.pmen.0000695 (PMC13496427; doi:10.1371/journal.pmen.0000695)
Supplement: Supplementary file 1 [file pmen.0000695.s003.pdf]

We would like to thank all of the reviewers for taking the time to provide us with some detailed and helpful feedback on our paper. We have outline below how we have aimed to address each point and we believe that the paper is much stronger now as a results of these suggestions.

**Reviewer One**	
1. The authors point out "systemic limitations" and "fragmented services," but they do not explain how new technologies, like secure data sharing and AI monitoring, could help solve these issues. The current discussion of solutions is not specific enough.	Thank you for taking time to review our paper, and for the thoughtful suggestions on how we may improve it. We have now added sections into our discussion chapter that integrates the use of data sharing and the challenges and benefits that may come with this for supporting parents of children with send.
2. The authors define support mainly in traditional terms, focusing on General Practice (GP) and Child and Adolescent Mental Health Services (CAMHS). They do not consider digital health tools or automated monitoring systems.	Thank you for this suggestion. The parents in our study did not mention digital tools which, in itself, is of interest. We have now added a section into the discussion to explore why this may be, and how digital tools or AI might be used to support mental health in this cohort:<br><br>Accessing traditional forms of mental health support was a challenge for this cohort, partially because of the complexities of arranging childcare in order to go to in-person appointments. One alternative may be to promote digital mental health support tools. It is unlikely that, given that parents often seek support informally due to the challenges highlighted above, they have information about access to digital tools. Digital mental health care has the potential to make prevention, diagnosis, and treatment of mental illness flexible, cost-effective, and accessible, regardless of

	location (Paganini et al., 2018; Spanhel et al., 2021). Tools such as rule-based and generative AI chatbots have been shown to be supportive for those with mental health difficulties (Siddals et al., 2024), and their round-the-clock availability and accessibility via mobile phones allow individuals to obtain help at any time or place, overcoming financial and logistical barriers, which would be beneficial for parents of SEND globally. The limitations of these tools must be clearly explained to users; however, there is limited research exploring their acceptability among parents of SEND or within the wider neurodivergent population. Future research would benefit from further exploration of their potential to support this cohort's mental health needs and the adaptations needed to make the tools usable for diverse needs.
3. The study is limited to York, England. The authors should explain how these findings can be applied to other areas or regulatory settings.	We have now integrated more information throughout the paper on how the findings here may, or may not, be applicable to other settings or geographical areas.
4. The survey data includes demographics and awareness levels, but there is little analysis of how specific demographics, such as child	As our current analysis did not aim to look at subgroups in such detail, we have chosen not to include this in the current paper, however, we do agree that this is a limitation of our analysis that should be included in future work and have addressed this in our

diagnosis type, relate to lower service awareness or satisfaction.	limitations section:<br><br>Finally, no information on ethnicity or deprivation/socioeconomic status was collected during this study, meaning that intersectionality and links between potential inequalities and the accessibility of mental health services cannot be discussed. Future research would benefit from gathering this additional information to explore which families may need further support accessing services, including exploring child diagnosis type, service awareness and satisfaction.
5. In Introduction or Discussion (Future Directions). It is suggested to refer from the paper: “Mental Health Monitoring and Intervention Using Unsupervised Deep Learning on EEG Data “. This reference provides insights into new methods for monitoring mental health using deep learning and EEG technology. Citing this work strengthens the manuscript by comparing the current manual systems with future options for effective and non-invasive monitoring tools. These advancements could ease the burden on parents by simplifying assessments and providing "innovative solutions" for mental health support.	As our current study is guided by the experiences of parents, we have chosen not to include a deep discussion on the use of these technologies within the paper. However, we do see how advancements in this area could benefit this population, and have added a paragraph in our limitations section that reflects this:<br><br>As this paper focused on parents' experiences, their suggestions on how future support should be shaped may be limited by the services they are aware of or have accessed in the past. Future work should explore the acceptability of new methods for monitoring mental health, such as those using EEG or deep learning, and how advancements in digital tools may

	provide mental health support solutions that ease the burden on parents.
6. The authors discuss the need for “joined-up” services but need to expand on how modern health data systems, like blockchain and secure data sharing, can help. The lack of communication between services is a problem with data, not just policy.	Thank you for this suggestion. The role of such technology and the need for data sharing has now been discussed:<br><br>In addition to a family-focused approach to support, parents desired a wraparound style of services that integrate and communicate with one another, and more practical support, such as skilled childcare. While there is a clear need for this, as it would reduce the administrative burden on parents, it would require improvements in data capture and data sharing between services. Studies have shown that blockchain can improve healthcare services by enabling the secure sharing of sensitive data (Taherdoost, 2023), but a wider discussion of this is beyond the scope of the current study. Future studies should explore the role that secure data sharing could play in improving SEND support.
7. The literature review focuses too much on psychological effects. It should also include recent studies on healthcare optimization and new diagnostic and monitoring tools to better explain the "future of support."	We have significantly revised our introduction section and hope that the restructuring and addition of new literature now helps to frame our suggestions for future support.

8. The authors state, "Research data are not shared due to ethical restrictions." They should acknowledge that privacy issues are a major barrier to integrating services and refer to relevant literature on privacy in healthcare.	We have aimed to cover this suggestion in point 6, where we discuss data sharing.
**Reviewer Two**	
I would like to thank the authors for conducting this important study. Below, I have outlined some suggestions that I think potentially can improve the presentation.	Thank you for your suggestions below, we hope that we have addressed your comments thoughtfully and sufficiently.
I was somewhat confused in the abstracts regarding the difference between the survey and the interviews. After reading the abstract, I expected that the study would constitute two parts, one quantitative part based on the survey, and one qualitative part based on the interviews. However, the survey appears to have been used to collect qualitative information and I that this is clarified early on.	Thank you for this suggestion, we have now made it clear in the abstract that the data from both methods were qualitative and now reads:<br><br>Qualitative data from 66 survey respondents (89.4% female) and 12 interviewees (67% female) were thematically analysed.
On page 4, line 87-88, the lack of support is argued to increase mental health difficulties. However, in other parts of the manuscript, mental health support is considered to reduce mental health problems. Thus, there is a discrepancy between how support is viewed – is it the norm (and lack causes poor mental health) or is support an intervention that impacts the "natural progression" of mental health problems of having a child with SEND?	We have now revised the whole introduction to ensure that the structure is clearer. In doing so, we hope that we have addressed this discrepancy.
I would suggest the authors to review the aim and research questions in relation to the results. The aim focus on if parents are "awareness of mental health services", while the research questions focus on "need of mental health services". The result section presents issues not obviously covered in the aim/RQ, such as the	We have now updated the wording in the aims section to better reflect the aims of the paper, and we have revisited the results section to ensure that our findings are more clearly linked to the research questions we are exploring.

how parental mental health is affected by the child and the problems of "navigating the complexity of SEND family life". I think the presentation would benefit from a more stringent link aim – research question – results.	
Page 14, line 286: It is not clear how the authors study "self-esteem" or how this is linked to mental health.	Thank you for spotting this error. This has now been corrected to reflect the self-identity that the participants were referring to:<br><br>This may suggest that even for those parents who are not explicitly speaking of isolation and loneliness, the demands of parenting could have a lasting impact on self-identity and, in turn, mental health.
I think the presentation would benefit from a clearer distinction between support services for the children and the support services for the parents – especially in the result presentation. As I understand, the purpose of the study is the latter, while the former is need factor/barrier to support for parents - and this should be clear in both the result and discussion sections.	Thank you for this suggestion. We have now restructured the results, with significant restructuring to theme three, to make it clear where we are talking about services for parents vs those that are aimed at supporting children. We have also aimed to make this much clearer throughout the discussion section using similar restructuring and signposting.
The discussion brings up the need for improved support in the transition between school and workplace, but I cannot see this being represented in the result section.	Thank you for spotting this error. We have now added a section in the results that reflects the call for services that support the transition into adult services, and the implication this has on parental mental health:<br><br>Furthermore, alongside wraparound care, joined-up support for children, extending into the transition to adulthood, was called for throughout the study. Parents reported that supporting their children did not stop when they

	turned 18, and highlighted the challenges that accompanied the switch to adult services, which often required multiple repetitions of admin that added pressure on parents and young people:<br><br>‘Every separate service you engage with, you have to get your young person's permission once they're over 18, or over 16 possibly, to talk to the service on their behalf. So I have that that for PIP, […] But if I want to talk to the GP on her behalf, she's got to come in with me, which she hates doing, and then talking to people she hates doing. […] And then the same with the ADHD service at the [place], and they’re like, “Well, your young person will need to come on the phone and tell us that we can talk to you” and I do get that it's really important that they do that. I do get that. But it would be really nice if I could just do that once and then it carried over to all of the other services, so I could sort it at the GP and then it would apply it to the hospital and it would apply to the [place], and you know, it's all a faff’ (Grace)<br><br>Services that could reduce the admin burden during key transition periods, and that take into account the communication needs and reasonable

	adjustments of young people, would, in turn, support parental mental health.
The authors appear to use “mental health” and “well-being” interchangeably, especially in the discussion section. This raises the question how mental health is defined in the study and how it is considered to be connected to (overall?) well-being.	Thank you for spotting this error. We have been through and updated the terminology to reflect that we are focusing on mental health, and have provided a definition for mental health and how we believe it links to wider well-being in a note on the first page:<br><br>For the purpose of this article, the authors adopt the definition of mental health provided by the World Health Organisation (2025): “Mental health is a state of mental well-being that enables people to cope with the stresses of life, realize their abilities, learn and work well, and contribute to their community. It has intrinsic and instrumental value and is a basic human right”. The authors see mental health as a continuum, and that individuals may be at risk of developing mental health disorders, such as depression or anxiety, which may require treatment and may impact on an individual’s wider well-being which encompasses the way they feel about their wider quality of life and their ability to contribute to society.
In the limitation, the authors discuss the possibility of skewed sample (compared to the population of parents with children with SEND) due to that the ones with greatest support needs opt out due to time constraints. I agree with this, but the alternative should also be acknowledged – it is potentially primarily those who are less satisfied with the existing support structures and/or those with most problems that are motivated to participate in the study. Without access to information on the full population, it is likely impossible to predict the direction of the bias.	We have now updated this section to reflect this suggestion:<br><br>Another limitation is that, although the study aimed to reach as many parents as possible through physical flyers and social media, it remains possible that those with the greatest support needs could not participate due to the additional time involved in the survey or interview. Alternatively, there is a risk that parents who are least satisfied with the existing support structures, or those who have the most difficulty accessing support, were more motivated to participate in the current study, which may

	further skew the sample. Without more detailed background information on participants, we are unable to identify which bias is more likely. Future work should focus on reducing this barrier by conducting work within the community, preferably in a setting where parents can bring their children if needed, and in a way that facilitates discussions for all parents, regardless of their current or previous engagement with services. Researchers should also offer flexible and multimodal ways to participate to make this easier for all community members.
The study is firmly set in the context of York, which the authors stress. However, a reader of an international journal would expect help with understanding if the results can be extrapolated (to England, UK, Europe, the world?). That is, I suggest that the authors explain to what extent the existing support structure is similar/different between York and other parts of England/UK and thereby if the results can be considered to be relevant also for other areas. Also highlight the limit of the extrapolation.	We have now integrated how the findings may or may not be generalisable to different areas of the UK, and globally, throughout the discussion. We have added a specific discussion point on this, and addressed the challenges with extrapolation in the limitations section.
Page 4, line 86: “lack of awareness” of what?	Please see comment above.
Figures and tables should be self-explaining (explain abbreviations)	Abbreviations for all tables and figures has now been added.
What is the value of table 2? Consider removing/condensing	We have removed the table and included this in supplementary materials for those who are interested in this information.
**Reviewer Three**	

This paper describes a qualitative study investigating an important and timely issue, namely mental health support for parents of children with special educational needs and disabilities. It is well-written and clearly describes the key findings related to this complex issue. I have a few specific comments that I think would strengthen this paper.	Thank you for your suggestions, we hope that below we have addressed your concerns and suggestions sufficiently.
Method<br><br>On pp. 6-9, the demographics for the survey and interview samples are described, but there do not appear to be any data on ethnicity or deprivation/SES. If this is the case, can we assess inclusivity and representativeness of the sample? Is there any way to get a sense of this? This information would provide important clues about potential inequalities in access to mental health support for parents. If this isn't possible, or if this information is limited, this should be acknowledged in the limitations section.	Thank you for raising this limitation. To limit the amount of data we collected from parents, we did not ask for information on ethnicity or SES. We did collect the first half of postcodes to check the location of participants were eligible to take part in the study, however, as deprivation can only be calculated using full postcodes (and we did not collect this to limit identifying participants), we are unable to calculate this. We agree this is a limitation of the study and have now added the following to limitations:<br><br>Finally, no information on ethnicity or deprivation/socioeconomic status was collected during this study, meaning that intersectionality and links between potential inequalities and the accessibility of mental health services cannot be discussed. Future research would benefit from gathering this additional information to explore which families may need further support accessing services.
On p.8, lines 174-177, more information is needed about whether there were any selection criteria or targeting used to select participants for interviews from the survey sample or via other recruitment.	Thank you for this suggestion. We have now added the following to our methods section:<br><br>For those not recruited through the survey, the same selection criteria were

	used. Targeted recruitment was conducted through a local support group for men to ensure a more balanced gender split in the interview study.
Discussion<br><br>The discussion focuses primarily, probably rightly, on the prevention of mental health problems in parents of children with SEND by discussing the findings related to improving support for children and the family as a whole. Although an emphasis on this is justified, there is perhaps not enough reflection in the discussion section on the need for tailored mental health support from consistent professionals who have training or lived experience of SEND, provided in ways that make it accessible to parents of children with SEND, including support to feel safe to seek help, as described in the results.	We agree with this suggestion and, as a result, have significantly revised the discussion to ensure it focuses more firmly on tailored mental health for parents.
On p. 22, lines 506-513 describe findings that I do not believe are reported in the results section, although they do relate to an important point about the long-term nature of caring for a child with SEND, which is different from the more common family caring role of caring for elderly relatives over a shorter time period, as well as the need for support for parent carers at transition points.	Thank you for highlighting this error. We have now added a section in the results to support this (see comment above for reviewer 2).
As mentioned above, the limitations section should reflect that this study may be lacking key demographics that would provide information about potential inequalities and how intersectionality may affect access to support for parents of children with SEND.	Please see comment above.
