# Supplementary material for: Experiences of mental health support services in a community sample of parents of children with special educational needs and disabilities
Source: PLOS Ment Health. 2026 Aug 21;3(8):e0000695. doi: 10.1371/journal.pmen.0000695 (PMC13496427; doi:10.1371/journal.pmen.0000695)
Supplement: Supplementary file 2 [file pmen.0000695.s004.pdf]

Reviewer 2 Comment	Response
**Reviewer #2:** I believe that the revision has greatly improved the paper, especially in terms of the alignment between aim and results, and in terms of distinguishing between support for parents and support for children, with the beneficial effect that this is expected to have for parents. I have however two further comments on issues that have become more apparent with the added clarity of the revision.	Thank you for taking the time to review our revision of the paper, and for your helpful and thoughtful suggestions. We have highlighted below how we have addressed each point.
The introduction firmly establishes that parents to children with SEND have poor mental health and restricted access to support and services (see for example the first paragraph and the third page (lines 107-131)). In light of this, I would encourage the authors to help the reader, in the introduction, to understand what knowledge gap this particular study tries to fill and how the reader should understand the contribution of the study.	Thank you for this suggestion; we have been through the introduction and signposted the reader to the gaps in the current research and how the current study aims to build on previous work.
Given prior research, what is the contribution of this study; why conduct a study to investigate this in York when it is already "known" in prior research? Connected to this is if the paper is specifically about the situation in York, or if the paper is performed in York but trying to say something about the situation in England/UK. I understand the current writing as the former, which raises the question on why the situation in York is of such particular interest? (I believe that it would be possible to argue the latter based on the shared policy framework within the country and the similarity of the NHS across regions.)	We have now added a section at the end of the introduction which helps to describe York as a city, and situate it within the wider context of NHS ICSs and the workings of local education authorities:<br><br>*The current study*<br><br>Previous research clearly shows that parents of children with SEND are not only at risk of poorer mental health, but that they have restricted access to suitable support services; however, current research focuses predominantly on small

	groups of parents supporting specific SEND (e.g., autism). The current study aims to expand this knowledge by exploring the experiences of parents with children who have a wide range of needs in more detail in the specific context of York. York serves as an excellent micro-system for studying parents' mental health experiences when caring for children with special educational needs and disabilities (SEND), providing insights that are unique to York and that can be directly applied across the UK. Systemically, parents in York navigate the standard institutional frameworks and structural pressures characteristic of English local authorities and NHS integrated care systems, including Education, Health and Care Plan (EHCP) pathways, multi-agency coordination, and delays in specialised services. Furthermore, whilst York's socio-economic landscape mirrors broader national health divides, featuring pronounced variations between affluent suburban wards and localised pockets of socioeconomic deprivation, around 5% of York's population live in areas ranked amongst the bottom fifth in England (City of York Council, 2023), placing areas of the city in the NHS Core20Plus5 (NHS, N.D.). This has led to wide inequalities in healthcare access, and allowing the exploration of how local support networks and community support in different wealth areas may influence parental mental health and access to support.

	While localised commissioning structures may vary slightly by region, the fundamental stressors and systemic barriers experienced by families raising children with special needs remain structurally consistent across England. Consequently, findings from York can yield scalable, nationally relevant insights capable of informing family-centred mental health policies, early intervention strategies, and targeted support frameworks for SEND caregivers across the UK. Therefore, using a combination of survey and interview methods, the current study aimed to develop an understanding of parents' and caregivers' mental health and their experiences of navigating services in the City of York and the surrounding areas, including whether current services are perceived to be adequately meeting their needs, and how parents believe that service provision could be improved. The research questions were:
There are still some statements in the discussion that appear not based on the results, alternatively not shown in the result section. For example the distinction between diagnostic label and need profiles. I would suggest that the authors double check that all statements in the discussion is based on the results, as well as written out in the result section.	Thank you for highlighting this. We have been through the discussion and ensured that claims made align with what was reported in the results section. Where they were not supported by the results directly, we reworded points to ensure they read as a recommendation/interpretation of the research team, and not phrased in a way that suggests our participants directly reported them.
Abstract: Explain the SEND abbreviation	We have explained this abbreviation in the abstract.

Lines 80-81: Wording seems a bit off.	Thank you for highlighting this, we have now restructured the sentence to read:<br><br>This suggests a need for support services to work at the family level and to take into account the multifaceted challenges parents encounter, rather than working with a single parent in isolation.
There are some abbreviations in the discussion section that are not explained.	We have reviewed the discussion section and ensured all abbreviations are explained and highlighted in the manuscript.
