# Supplementary material for: Experiences of mental health support services in a community sample of parents of children with special educational needs and disabilities
Source: PLOS Ment Health. 2026 Aug 21;3(8):e0000695. doi: 10.1371/journal.pmen.0000695 (PMC13496427; doi:10.1371/journal.pmen.0000695)
Supplement: Supplementary file 3 [file pmen.0000695.s001.docx]

**S1 Table**

Demographic information for interview participants with pseudonyms.

| **Pseudonym** | **Sex** | **Age** | **Marital Status** |
| --- | --- | --- | --- |
| **Mary** | Female | 37 | Cohabiting |
| **James** | Male | 54 | Married |
| **Rachel** | Female | 48 | Married |
| **Victoria** | Female | 49 | Married |
| **Hannah** | Female | 51 | Married |
| **Grace** | Female | 55 | Single |
| **Emma** | Female | 46 | Married |
| **David** | Male | 57 | Married |
| **Julia** | Female | 55 | Single |
| **Sarah** | Female | 36 | Married |
| **Henry** | Male | 57 | Married |
| **Lucas** | Male | 43 | Married |
