# Supplementary material for: Experiences of mental health support services in a community sample of parents of children with special educational needs and disabilities
Source: PLOS Ment Health. 2026 Aug 21;3(8):e0000695. doi: 10.1371/journal.pmen.0000695 (PMC13496427; doi:10.1371/journal.pmen.0000695)
Supplement: Peer review history

# Peer review: Decision Letter 0

**DOI:** 10.1371/journal.pmen.0000695.r001

**Authors:** Vanessa Carels

20 Apr 2026

PMEN-D-25-00596

Experiences of Mental Health Support Services in a Community Sample of Parents of Children with Special Educational Needs and Disabilities

PLOS Mental Health

Dear Dr. Fox,

Thank you for submitting your manuscript to PLOS Mental Health. After careful consideration, we feel that it has merit but does not fully meet PLOS Mental Health’s publication criteria as it currently stands. Therefore, we invite you to submit a revised version of the manuscript that addresses the points raised during the review process.

Please submit your revised manuscript by Jun 04 2026 11:59PM. If you will need more time than this to complete your revisions, please reply to this message or contact the journal office at mentalhealth@plos.org. When you're ready to submit your revision, log on to https://www.editorialmanager.com/pmen/ and select the 'Submissions Needing Revision' folder to locate your manuscript file.

Please include the following items when submitting your revised manuscript:

- A letter that responds to each point raised by the editor and reviewer(s). You should upload this letter as a separate file labeled 'Response to Reviewers'.
- A marked-up copy of your manuscript that highlights changes made to the original version. You should upload this as a separate file labeled 'Revised Manuscript with Track Changes'.
- An unmarked version of your revised paper without tracked changes. You should upload this as a separate file labeled 'Manuscript'.

Guidelines for resubmitting your figure files are available below the reviewer comments at the end of this letter.

As the corresponding author, your ORCID iD is verified in the submission system and will appear in the published article. PLOS supports the use of ORCID, and we encourage all coauthors to register for an ORCID iD and use it as well. Please encourage your coauthors to verify their ORCID iD within the submission system before final acceptance, as unverified ORCID iDs will not appear in the published article. *Only*  the individual author can complete the verification step; PLOS staff *cannot*  verify ORCID iDs on behalf of authors.

We look forward to receiving your revised manuscript.

Kind regards,

Vanessa Carels

Staff Editor

PLOS Mental Health

**Journal Requirements:**

1. Please amend your detailed Financial Disclosure statement. This is published with the article. It must therefore be completed in full sentences and contain the exact wording you wish to be published.

i. Please clarify all sources of financial support for your study. List the grants, grant numbers, and organizations that funded your study, including funding received from your institution. Please note that suppliers of material support, including research materials, should be recognized in the Acknowledgements section rather than in the Financial Disclosure.

ii. State the initials, alongside each funding source, of each author to receive each grant. For example: "This work was supported by the National Institutes of Health (####### to AM; ###### to CJ) and the National Science Foundation (###### to AM)."

iii. State what role the funders took in the study. If the funders had no role in your study, please state: “The funders had no role in study design, data collection and analysis, decision to publish, or preparation of the manuscript.”

iv. If any authors received a salary from any of your funders, please state which authors and which funders.

2. We note that you have indicated that there are restrictions to data sharing for this study. For studies involving human research participant data or other sensitive data, we encourage authors to share de-identified or anonymized data. However, when data cannot be publicly shared for ethical reasons, we allow authors to make their data sets available upon request. For information on unacceptable data access restrictions, please see http://journals.plos.org/plosone/s/data-availability#loc-unacceptable-data-access-restrictions.

Before we proceed with your manuscript, please address the following prompts:

a) If there are ethical or legal restrictions on sharing a de-identified data set, please explain them in detail (e.g., data contain potentially identifying or sensitive patient information, data are owned by a third-party organization, etc.) and who has imposed them (e.g., a Research Ethics Committee or Institutional Review Board, etc.). Please also provide contact information for a data access committee, ethics committee, or other institutional body to which data requests may be sent.

b) If there are no restrictions, please upload the minimal anonymized data set necessary to replicate your study findings to a stable, public repository and provide us with the relevant URLs, DOIs, or accession numbers. Please see http://www.bmj.com/content/340/bmj.c181.long for guidelines on how to de-identify and prepare clinical data for publication. For a list of recommended repositories, please see https://journals.plos.org/plosone/s/recommended-repositories. You also have the option of uploading the data as Supporting Information files, but we would recommend depositing data directly to a data repository if possible.

Please update your Data Availability statement in the submission form accordingly.

3. Please ensure that your Ethics Statement is available in its entirety at the beginning of your Methods section, under a subheading 'Ethics Statement'.

4. Please upload separate figure files in .tif or .eps format. Also, remove the figures from your manuscript file but keep the legends.

For more information about figure files please see our guidelines:

https://journals.plos.org/mentalhealth/s/figures

https://journals.plos.org/mentalhealth/s/figures#loc-file-requirements

If the reviewer comments include a recommendation to cite specific previously published works, please review and evaluate these publications to determine whether they are relevant and should be cited. There is no requirement to cite these works unless the editor has indicated otherwise.

**Additional Editor Comments (if provided):**

[Note: HTML markup is below. Please do not edit.]

Reviewers' comments:

Reviewer's Responses to Questions

**Comments to the Author**

1. Does this manuscript meet PLOS Mental Health’s publication criteria?

Reviewer #1: Yes

Reviewer #2: Partly

Reviewer #3: Yes

**********

2. Has the statistical analysis been performed appropriately and rigorously?

Reviewer #1: Yes

Reviewer #2: N/A

Reviewer #3: N/A

**********

3. Have the authors made all data underlying the findings in their manuscript fully available (please refer to the Data Availability Statement at the start of the manuscript PDF file)?

The PLOS Data policy

Reviewer #1: Yes

Reviewer #2: No

Reviewer #3: Yes

**********

4. Is the manuscript presented in an intelligible fashion and written in standard English?

Reviewer #1: Yes

Reviewer #2: Yes

Reviewer #3: Yes

**********

**Reviewer #1:**  1. The authors point out "systemic limitations" and "fragmented services," but they do not explain how new technologies, like secure data sharing and AI monitoring, could help solve these issues. The current discussion of solutions is not specific enough.

2. The authors define support mainly in traditional terms, focusing on General Practice (GP) and Child and Adolescent Mental Health Services (CAMHS). They do not consider digital health tools or automated monitoring systems.

3. The study is limited to York, England. The authors should explain how these findings can be applied to other areas or regulatory settings.

4. The survey data includes demographics and awareness levels, but there is little analysis of how specific demographics, such as child diagnosis type, relate to lower service awareness or satisfaction.

5. In Introduction or Discussion (Future Directions). It is suggested to refer from the paper: “Mental Health Monitoring and Intervention Using Unsupervised Deep Learning on EEG Data “. This reference provides insights into new methods for monitoring mental health using deep learning and EEG technology. Citing this work strengthens the manuscript by comparing the current manual systems with future options for effective and non-invasive monitoring tools. These advancements could ease the burden on parents by simplifying assessments and providing "innovative solutions" for mental health support.

6. The authors discuss the need for “joined-up” services but need to expand on how modern health data systems, like blockchain and secure data sharing, can help. The lack of communication between services is a problem with data, not just policy.

7. The literature review focuses too much on psychological effects. It should also include recent studies on healthcare optimization and new diagnostic and monitoring tools to better explain the "future of support."

8. The authors state, "Research data are not shared due to ethical restrictions." They should acknowledge that privacy issues are a major barrier to integrating services and refer to relevant literature on privacy in healthcare.

**Reviewer #2:**  I would like to thank the authors for conducting this important study. Below, I have outlined some suggestions that I think potentially can improve the presentation.

I was somewhat confused in the abstracts regarding the difference between the survey and the interviews. After reading the abstract, I expected that the study would constitute two parts, one quantitative part based on the survey, and one qualitative part based on the interviews. However, the survey appears to have been used to collect qualitative information and I that this is clarified early on.

On page 4, line 87-88, the lack of support is argued to increase mental health difficulties. However, in other parts of the manuscript, mental health support is considered to reduce mental health problems. Thus, there is a discrepancy between how support is viewed – is it the norm (and lack causes poor mental health) or is support an intervention that impacts the “natural progression” of mental health problems of having a child with SEND?

I would suggest the authors to review the aim and research questions in relation to the results. The aim focus on if parents are “awareness of mental health services”, while the research questions focus on “need of mental health services”. The result section presents issues not obviously covered in the aim/RQ, such as the how parental mental health is affected by the child and the problems of “navigating the complexity of SEND family life”. I think the presentation would benefit from a more stringent link aim – research question – results.

Page 14, line 286: It is not clear how the authors study “self-esteem” or how this is linked to mental health.

I think the presentation would benefit from a clearer distinction between support services for the children and the support services for the parents – especially in the result presentation. As I understand, the purpose of the study is the latter, while the former is need factor/barrier to support for parents - and this should be clear in both the result and discussion sections.

The discussion brings up the need for improved support in the transition between school and workplace, but I cannot see this being represented in the result section.

The authors appear to use “mental health” and “well-being” interchangeably, especially in the discussion section. This raises the question how mental health is defined in the study and how it is considered to be connected to (overall?) well-being.

In the limitation, the authors discuss the possibility of skewed sample (compared to the population of parents with children with SEND) due to that the ones with greatest support needs opt out due to time constraints. I agree with this, but the alternative should also be acknowledged – it is potentially primarily those who are less satisfied with the existing support structures and/or those with most problems that are motivated to participate in the study. Without access to information on the full population, it is likely impossible to predict the direction of the bias.

The study is firmly set in the context of York, which the authors stress. However, a reader of an international journal would expect help with understanding if the results can be extrapolated (to England, UK, Europe, the world?). That is, I suggest that the authors explain to what extent the existing support structure is similar/different between York and other parts of England/UK and thereby if the results can be considered to be relevant also for other areas. Also highlight the limit of the extrapolation.

Page 4, line 86: “lack of awareness” of what?

Figures and tables should be self-explaining (explain abbreviations)

What is the value of table 2? Consider removing/condensing

**Reviewer #3:**  This paper describes a qualitative study investigating an important and timely issue, namely mental health support for parents of children with special educational needs and disabilities. It is well-written and clearly describes the key findings related to this complex issue. I have a few specific comments that I think would strengthen this paper.

Method

On pp. 6-9, the demographics for the survey and interview samples are described, but there do not appear to be any data on ethnicity or deprivation/SES. If this is the case, can we assess inclusivity and representativeness of the sample? Is there any way to get a sense of this? This information would provide important clues about potential inequalities in access to mental health support for parents. If this isn't possible, or if this information is limited, this should be acknowledged in the limitations section.

On p.8, lines 174-177, more information is needed about whether there were any selection criteria or targeting used to select participants for interviews from the survey sample or via other recruitment.

Discussion

The discussion focuses primarily, probably rightly, on the prevention of mental health problems in parents of children with SEND by discussing the findings related to improving support for children and the family as a whole. Although an emphasis on this is justified, there is perhaps not enough reflection in the discussion section on the need for tailored mental health support from consistent professionals who have training or lived experience of SEND, provided in ways that make it accessible to parents of children with SEND, including support to feel safe to seek help, as described in the results.

On p. 22, lines 506-513 describe findings that I do not believe are reported in the results section, although they do relate to an important point about the long-term nature of caring for a child with SEND, which is different from the more common family caring role of caring for elderly relatives over a shorter time period, as well as the need for support for parent carers at transition points.

As mentioned above, the limitations section should reflect that this study may be lacking key demographics that would provide information about potential inequalities and how intersectionality may affect access to support for parents of children with SEND.

**********

what does this mean?). If published, this will include your full peer review and any attached files.

**Do you want your identity to be public for this peer review?**  If you choose “no”, your identity will remain anonymous but your review may still be made public.

For information about this choice, including consent withdrawal, please see our Privacy Policy

Reviewer #1: No

Reviewer #2: No

Reviewer #3: No

**********

[NOTE: If reviewer comments were submitted as an attachment file, they will be attached to this email and accessible via the submission site. Please log into your account, locate the manuscript record, and check for the action link "View Attachments". If this link does not appear, there are no attachment files.]

**Figure Resubmissions:**

# Author comment: Author response to Decision Letter 1

**DOI:** 10.1371/journal.pmen.0000695.r002

23 Jun 2026

Attachment Submitted filename: Response to reviewer comments PLOS June 2026.pdf

# Peer review: Decision Letter 1

**DOI:** 10.1371/journal.pmen.0000695.r003

**Authors:** Olga Dobrushina

13 Jul 2026

PMEN-D-25-00596R1

Experiences of Mental Health Support Services in a Community Sample of Parents of Children with Special Educational Needs and Disabilities

PLOS Mental Health

Dear Dr. Fox,

Thank you for submitting your manuscript to PLOS Mental Health. After careful consideration, we feel that it has improved significantly but would benefit from a few minor edits. Therefore, we invite you to submit a revised version of the manuscript that addresses the points raised during the review process.

The revision comprehensively addresses the reviewers' comments, and the paper is in very good shape overall. Please address the remaining minor comments before the article can be accepted.

Please submit your revised manuscript by Aug 12 2026 11:59PM. If you will need more time than this to complete your revisions, please reply to this message or contact the journal office at mentalhealth@plos.org. When you're ready to submit your revision, log on to https://www.editorialmanager.com/pmen/ and select the 'Submissions Needing Revision' folder to locate your manuscript file.

Please include the following items when submitting your revised manuscript:

- A letter that responds to each point raised by the editor and reviewer(s). You should upload this letter as a separate file labeled 'Response to Reviewers'.
- A marked-up copy of your manuscript that highlights changes made to the original version. You should upload this as a separate file labeled 'Revised Manuscript with Track Changes'.
- An unmarked version of your revised paper without tracked changes. You should upload this as a separate file labeled 'Manuscript'.

Guidelines for resubmitting your figure files are available below the reviewer comments at the end of this letter.

As the corresponding author, your ORCID iD is verified in the submission system and will appear in the published article. PLOS supports the use of ORCID, and we encourage all coauthors to register for an ORCID iD and use it as well. Please encourage your coauthors to verify their ORCID iD within the submission system before final acceptance, as unverified ORCID iDs will not appear in the published article. *Only*  the individual author can complete the verification step; PLOS staff *cannot*  verify ORCID iDs on behalf of authors.

We look forward to receiving your revised manuscript.

Kind regards,

Olga Dobrushina, MD, PhD

Academic Editor

PLOS Mental Health

**Journal Requirements:**

If the reviewer comments include a recommendation to cite specific previously published works, please review and evaluate these publications to determine whether they are relevant and should be cited. There is no requirement to cite these works unless the editor has indicated otherwise.

Please review your reference list to ensure that it is complete and correct. If you have cited papers that have been retracted, please include the rationale for doing so in the manuscript text, or remove these references and replace them with relevant current references. Any changes to the reference list should be mentioned in the rebuttal letter that accompanies your revised manuscript. If you need to cite a retracted article, indicate the article’s retracted status in the References list and also include a citation and full reference for the retraction notice.

[Note: HTML markup is below. Please do not edit.]

Reviewers' comments:

Reviewer's Responses to Questions

**Comments to the Author**

Reviewer #2: (No Response)

**********

publication criteria?

Reviewer #2: Partly

**********

3. Has the statistical analysis been performed appropriately and rigorously?

Reviewer #2: N/A

**********

4. Have the authors made all data underlying the findings in their manuscript fully available (please refer to the Data Availability Statement at the start of the manuscript PDF file)?

The PLOS Data policy

Reviewer #2: No

**********

5. Is the manuscript presented in an intelligible fashion and written in standard English?

Reviewer #2: Yes

**********

**Reviewer #2:**  I believe that the revision has greatly improved the paper, especially in terms of the alignment between aim and results, and in terms of distinguishing between support for parents and support for children, with the beneficial effect that this is expected to have for parents. I have however two further comments on issues that have become more apparent with the added clarity of the revision.

Framing of the paper/aim

The introduction firmly establishes that parents to children with SEND have poor mental health and restricted access to support and services (see for example the first paragraph and the third page (lines 107-131)). In light of this, I would encourage the authors to help the reader, in the introduction, to understand what knowledge gap this particular study tries to fill and how the reader should understand the contribution of the study.

Given prior research, what is the contribution of this study; why conduct a study to investigate this in York when it is already “known” in prior research? Connected to this is if the paper is specifically about the situation in York, or if the paper is performed in York but trying to say something about the situation in England/UK. I understand the current writing as the former, which raises the question on why the situation in York is of such particular interest? (I believe that it would be possible to argue the latter based on the shared policy framework within the country and the similarity of the NHS across regions.)

Discussion

There are still some statements in the discussion that appear not based on the results, alternatively not shown in the result section. For example the distinction between diagnostic label and need profiles. I would suggest that the authors double check that all statements in the discussion is based on the results, as well as written out in the result section.

Minor

Abstract: Explain the SEND abbreviation

Lines 80-81: Wording seems a bit off.

There are some abbreviations in the discussion section that are not explained.

**********

what does this mean?). If published, this will include your full peer review and any attached files.

**Do you want your identity to be public for this peer review?**  If you choose “no”, your identity will remain anonymous but your review may still be made public.

For information about this choice, including consent withdrawal, please see our Privacy Policy

Reviewer #2: No

**********

[NOTE: If reviewer comments were submitted as an attachment file, they will be attached to this email and accessible via the submission site. Please log into your account, locate the manuscript record, and check for the action link "View Attachments". If this link does not appear, there are no attachment files.]

**Figure Resubmissions:**

# Author comment: Author response to Decision Letter 2

**DOI:** 10.1371/journal.pmen.0000695.r004

27 Jul 2026

Attachment Submitted filename: Response to reviewer comments R2.pdf

# Editor report: Decision Letter 2

**DOI:** 10.1371/journal.pmen.0000695.r005

**Authors:** Olga Dobrushina

30 Jul 2026

Experiences of Mental Health Support Services in a Community Sample of Parents of Children with Special Educational Needs and Disabilities

PMEN-D-25-00596R2

Dear Dr Fox,

We are pleased to inform you that your manuscript 'Experiences of Mental Health Support Services in a Community Sample of Parents of Children with Special Educational Needs and Disabilities' has been provisionally accepted for publication in PLOS Mental Health.

Before your manuscript can be formally accepted you will need to complete some formatting changes, which you will receive in a follow up email. A member of our team will be in touch with a set of requests.

Please note that your manuscript will not be scheduled for publication until you have made the required changes, so a swift response is appreciated.

IMPORTANT: The editorial review process is now complete. PLOS will only permit corrections to spelling, formatting or significant scientific errors from this point onwards. Requests for major changes, or any which affect the scientific understanding of your work, will cause delays to the publication date of your manuscript.

If your institution or institutions have a press office, please notify them about your upcoming paper to help maximize its impact. If they'll be preparing press materials, please inform our press team as soon as possible -- no later than 48 hours after receiving the formal acceptance. Your manuscript will remain under strict press embargo until 2 pm Eastern Time on the date of publication. For more information, please contact mentalhealth@plos.org.

Thank you again for supporting Open Access publishing; we are looking forward to publishing your work in PLOS Mental Health.

Best regards,

Olga Dobrushina, MD, PhD

Academic Editor

PLOS Mental Health

***********************************************************

Reviewer Comments (if any, and for reference):
